# Local IgE in patients with allergic rhinitis

**DOI:** 10.1186/1939-4551-8-S1-A105

**Published:** 2015-04-08

**Authors:** Adriana Teixeira Rodrigues, Edecio Cunha Neto, Jorge Kalil, Fabio FM Castro, Clovis ES Galvão

**Affiliations:** 1 Hospital Das Clinicas, University of São Paulo, School of Medicine, Brazil

## Background

To evaluate the presence of total IgE and specific IgE for *Dermatophagoides pteronissinus* (Der p) in nasal lavage of patients with moderate to severe persistent allergic rhinitis, with or without intermittent asthma. Also, to correlate the levels of total IgE and specific IgE for Der p in the serum and in the nasal lavage.

## Methods

We selected 48 patients with moderate to severe rhinitis (12 of them had intermittent asthma) sensitized to Der p without prior treatment with allergen specific immunotherapy. The protocol was approved by the ethics committee. All participants signed informed consent. Determination of total IgE and specific IgE for Der p1 (D1) and Der p2 (D2) were performed in serum and nasal lavage, using ImmunoCAP method. Non parametric tests were used in the statistical analysis.

## Results

The mean age was 35.1 years old (median 33.5 years, SD 11.82 years). The mean total serum IgE was 367UL (234UL median and SD 433.9 UL) and in nasal lavage it was 32.2 UL (median 30.63UL and SD 13.2), ranging from 16 to 73UL. The mean serum specific IgE for D1 and D2 was 42.3 and 31.7 UL respectively. The mean specific IgE in nasal lavage for D1 and D2 was 1.7UL and 1.43 UL respectively and ranged from 0.5 to 14.9 UL for D1 and 0.4 to 12.75UL for D2. There was no statistical difference between the groups with moderate to severe persistent rhinitis alone or associated with asthma. We have estimated the value of total IgE in the nasal lavage by the linear regression model (√nasal total IgE = 6,448 + 0,233 x √serum total IgE); and specific IgE to D1 in the nasal lavage (√nasal IgE to D1 = 0.751 + 0.535 x √serum IgE to D1); and the specific IgE in nasal lavage to D2 (√nasal IgE to D2 = 0.721 + 0.488 x √serum IgE to D2).

## Conclusions

It has been reported that 77% of allergic patients have nasal specific IgE. We have found nasal specific IgE in 100% of cases. In our case series we could estimate the value of total IgE and specific IgE in nasal lavage from the value found in the serum. However, there is no consensus in the literature about the values of total and specific IgE in nasal lavage in atopic patients.

